# Histological assessment of a novel de-epithelialization method for connective tissue grafts harvested from the palate. An experimental study in cadavers

**DOI:** 10.1007/s00784-024-05734-y

**Published:** 2024-05-28

**Authors:** Natalia Bara-Gaseni, Adria Jorba-Garcia, Javier Alberdi-Navarro, Rui Figueiredo, Jose-Javier Bara-Casaus

**Affiliations:** 1 Dental and Maxillofacial Institute. Hospital Universitari Sagrat Cor, Grupo Quirónsalud, Barcelona, Spain; 2 Department of Oral Surgery and Implantology, Faculty of Medicine and Health Sciences, University of Barcelona, C/ Feixa Llarga S/N; Pavelló Govern, 2a Planta, Despatx 2.9, L’Hospitalet de Llobregat, 08907 Barcelona, Spain; 3 Department of Stomatology II. School of Medicine and Nursing, University of the Basque Country (UPV/EHU), Leioa, Spain; 4 Faculty of Medicine and Health Sciences, University of Barcelona (Spain). Researcher at the IDIBELL Institute, Barcelona, Spain; 5 University Hospital of Mutua Terrassa, University of Barcelona, Terrassa, Spain

**Keywords:** Histology, Oral mucosa, Phenotype, Plastic surgery, Connective Tissue

## Abstract

**Objectives:**

This study aims to compare the histological outcomes of three distinct de-epithelialization methods used in (connective tissue grafts) CTG harvested from the palate.

**Materials and methods:**

An experimental study using nine cadaver head specimens was carried out to compare 3 different de-epithelialization techniques for CTG. Eighteen samples were randomly allocated to three study groups: bone scraper, diamond bur and extraoral removal with a scalpel. The main outcome variable was the graft surface percentage without epithelium remains. Additionally, the time employed, and the graft thickness were also measured.

**Results:**

Sixteen CTGs were analyzed. The extraoral scalpel group presented a total surface area with no epithelium of 58.84% (22.68) and a mean de-epithelialization time of 3.7 min; the intraoral diamond bur group had 88.24% (41.3) of the surface with no epithelium and took 1.455 min, and the intraoral bone scraper showed 97.98% (5.99) of surface without epithelium and a mean time of 0.815 min (*P* < 0.05). Histological analysis showed significant differences between the bone scraper and the extraoral group (*P* = 0.009).

**Conclusion:**

The de-epithelialization technique with a bone scraper seems to be the most effective and fastest de-epithelialization technique for CTG. These findings need to be confirmed in future clinical studies with larger samples.

**Clinical relevance:**

The use of bone scrapers, could be a simple, effective and fast technique to de-epithelialize connective tissue grafts harvested from the palatal area for both novice and experienced surgeons.

## Introduction

Connective tissue grafts (CTG) are considered by several authors [1–4] to be the gold standard technique for treating gingival or mucosal deficiencies. One of the most commonly used donor sites for CTGs is the posterior lateral region of ​​the palate. This zone has an orthokeratinized epithelium that covers a dense connective tissue (lamina propria) and a layer of fatty and glandular tissue (submucosa) of varying thickness [5, 6]. The epithelium has an approximate thickness of 0.1—0.5 mm and a CTG should be 1.0–1.5 mm to minimize the amount of adipose and glandular tissue [7].

Several techniques have been described to obtain a palatal CTG with the aim of reducing patient morbidity and complications like postoperative bleeding or donor site infections or dehiscence [4]. These approaches could be classified into two large groups: intraoral and extraoral de-epithelialization. On the one hand, extraoral de-epithelialization consists of harvesting a free gingival graft and performing an extraoral de-epithelialization using a blade [8]. On the other hand, intraoral de-epithelialization consists of a variety of incisions that leave the epithelium covering the donor site (i.e., trap door technique, single incision or parallel incisions) [9–11]. Recently, a new technique that uses a high-speed diamond bur with irrigation to de-epithelialize the graft has been described [1, 4, 12].

Even though epithelium remnants in CTGs do not seem to compromise the clinical success in root coverage procedures [12], some authors have stated that these remnants may be associated with complications such as external root resorption or cysts [4, 13, 14]. Thus, to minimize any potential risk, total de-epithelialization of CTG is desirable [4]. However, the most suitable technique for de-epithelialization is still a matter of debate [4]. For this reason, further research into this topic seems to be necessary.

In dermatology, dermatomes are widely used for obtaining skin grafts [15]. However, in oral surgery and periodontology, the primary method for grafts harvesting is still the scalpel. It is noteworthy that bone scrapers could serve as an alternative for intraoral de-epithelialization of grafts on the palate, functioning similarly to dermatomes in the skin. To the best of the authors' knowledge, this approach for harvesting CTG from the posterior palate has not been previously described, and by employing this technique, clinicians can ensure a standardized width of epithelium removal, as the blade of the scraper consistently cuts at the same depth [15–17].

Thus, the main hypothesis of the present study was that bone scrapers can completely remove the epithelial layer of palatal soft tissue grafts in a faster and more straightforward way in comparison to other de-epithelialization techniques. Hence, the present research aims to evaluate the effectiveness of a new de-epithelialization method using a bone scraper for CTG harvested from the posterior area of the palate and compares it with an extraoral technique and the use of a diamond bur.

## Material and methods

### Study design

The study was carried out in accordance with the regulations for ex-vivo studies and was approved by the human subject’s ethics board of the Quirónsalud-Catalunya Hospital group (2023/36-CIR-HUSC) and was conducted in accordance with the Helsinki Declaration of 1975, as revised in 2013. The experimental phase of the study was conducted at the Faculty of Medicine and Health Sciences of the University of Barcelona (Barcelona, Spain) between January and July 2023.

The present study was designed as a randomized experimental study in human cadavers in which three de-epithelialization techniques for CTG harvested from the posterior area of the palate were evaluated. All human cadaver specimens were in a good state of conservation and did not present any lesions, signs of infection or inflammation in the palatal area. The following de-epithelialization techniques were assessed: Intraoral de-epithelialization with a bone scraper (BS group), intraoral de-epithelialization with a diamond bur (DB group) and extraoral de-epithelialization with a scalpel (ES group).

### Randomization and blinding

The randomization sequence was generated with STATA 14 software (StataCorp, College Station, TX, USA) by a blinded investigator. The treatment sequence and allocation of each donor site was concealed in opaque envelopes. The allocation ratio was 1:1:1. Due to the nature of the study, the operator could not be blinded (since different surgical techniques were used) but their allocation was not known until the start of the surgical procedure. The researcher that performed the histological analysis was blinded.

### Description of the intervention

**Fig. 1:**
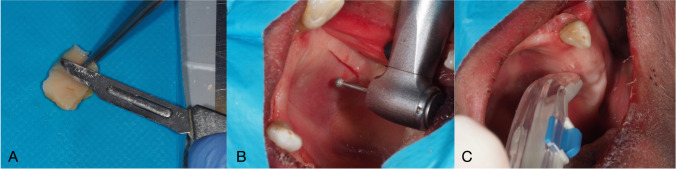
Clinical pictures of the de-epithelialization techniques. **A**. Extraoral de-epithelialization with scalpel; **B**. Intraoral de-epithelialization with a diamond bur; **C**. Intraoral de-epithelialization with a bone scraper

All grafts were harvested and de-epithelialized by the same operator (N.B.G). The operator was a first-year student in a postgraduate oral surgery program. All grafts were designed to be 15 mm length × 8 mm width and one graft was harvested from each hemiarch. The following surgical techniques were used (Fig. 1):

#### Extraoral technique with scalpel (ES technique)

With a 15C scalpel blade, a 1.0–1.5 mm thick and 15 mm long horizontal incision was done 2 mm apical from the gingival margin of the maxillary teeth. A parallel horizontal incision was made 8 mm apical to the first horizontal incision [1]. Then, both horizontal incisions were connected with two vertical incisions. Finally, the epithelialized palatal graft was harvested from the palate with a scalpel employing a split-thickness incision aiming to maintain a uniform graft thickness of 1.5 mm. The extraoral de-epithelialization was carried out with a new 15C blade, placing it parallel to the graft surface and irrigating with saline solution to facilitate smooth sliding of the scalpel blade and aiming to remove 0.5 mm thickness of the graft [8].

#### Intraoral technique with diamond bur (DB technique)

Four incisions (2 horizontal and 2 vertical) were made following the instructions given above for the extraoral technique. Before removing the tissue, a diamond ball bur at high speed (200,000 rpm) with sterile saline irrigation was used to remove the superficial layer (0.5 mm) of the graft [7]. Considering that the bur had a 1 mm diameter, the surgeon was instructed to deepen half of the bur in the palate. Once the de-epithelialization had been performed, the graft was harvested from the palate with a scalpel through a split-thickness incision aiming to maintain a uniform thickness of 1.5 mm [4].

#### Intraoral technique with bone scraper (BS technique)

Again, the same incisions as described earlier were performed. Afterwards, a bone scraper (Safescraper, curved twist model, META, Reggio Emilia, Italy) was used to remove the superficial layer of the graft (de-epithelialization). The bone scraper was applied 2 times over the graft to remove approximately 0.5mm. Once the de-epithelialization was performed, the graft was harvested from the palate with a scalpel using the previously described technique (split-thickness incision to obtain a uniform thickness of 1.5 mm).

### Histological analysis

All the samples were fixed in 10% buffered formalin for 24 h, in a volume of 20/1 with respect to the sample size. Before processing, a photographic record of the macroscopic appearance of the study samples was made. Prior to histopathological processing, the samples were sectioned longitudinally in their middle zone.

For the histopathological analysis, a conventional procedure of fixation, dehydration, paraffin embedding and staining with hematoxylin and eosin was performed. Dehydration using progressively increasing concentrations of alcohol solution was performed. Then inclusion in paraffin of the sample was performed taking special attention to achieve a proper orientation of the samples. All the samples were orientated by their cutting surface. Subsequently, sections of 5 μm were made and mounted on slides which were deparaffinized in an oven and with xylol solution. Then, hydration was carried out in decreasing alcohol concentrations ending with water (hydration and dehydration train). After that, staining with Gill® hematoxylin and eosin-phloxin was performed. A final dehydration was carried out in increasing alcohol concentrations. Finally, it was mounted in a permanent resinous medium.

Histopathological assessment was performed using an Olympus® optical microscope (BX51) with a magnification of × 20. Images were obtained in a calibrated manner with an Olympus® XC50 camera and Olympus® CellSens software.

### Study variables

The main outcome variable was the percentage of CTG surface with no epithelium remnants. A sagittal section of 5μm was obtained from the middle portion of each sample, then mounted onto a glass side and histopathologically analyzed. Calibrated images from all samples were obtained and imported to the ImageJ program (LOCI, University of Wisconsin, USA). To calculate the percentage of the surface without epithelium, the surface area with no epithelium remnants was divided by total surface area of the sample.

Additionally, a dichotomic outcome variable measuring the presence or absence of epithelial remnants in the samples was also recorded. Finally, the time needed for the de-epithelialization of the CTG (in minutes), the total thickness of the grafts (in mm), and the thickness of the remaining epithelium after de-epithelialization in the samples with epithelium remnants (in mm), was recorded.

### Statistical analysis

The sample calculation was carried out with the G* Power program version 3.1.9.2 (Universität Kiel, Germany). The calculation was made based on values from previous studies [4, 12] and assuming an alpha error of 0.05 and a beta power of 0.8. Using an ANOVA test and assuming that de-epithelialization rates would be of 80% for the extraoral scalpel, 84% for the bur technique and 95% for the bone scraper, with a standard deviation of 7, the sample calculation yielded a total of 18 grafts to be analyzed, 6 grafts per group.

A third blinded investigator performed the statistical analysis using STATA 14 software (StataCorp, College Station, TX, USA). A descriptive analysis of the data was performed. The normality of the scale variables was explored using the Shapiro-Wilks test and visual analysis of normal P-P plots and boxplots. Descriptive analysis using median and interquartile range (IQR) was used, since normality was rejected. The descriptive analysis of bivariate categorical variables was performed using absolute and relative frequency tables.

Subsequently, a multivariate analysis of the data was performed. A Kruskall Wallis test was used to compare the main response variables between the 3 groups. Subsequently, post hoc pair wise comparisons were explored. The significance level was set at 0.05.

## Results

**Fig. 2:**
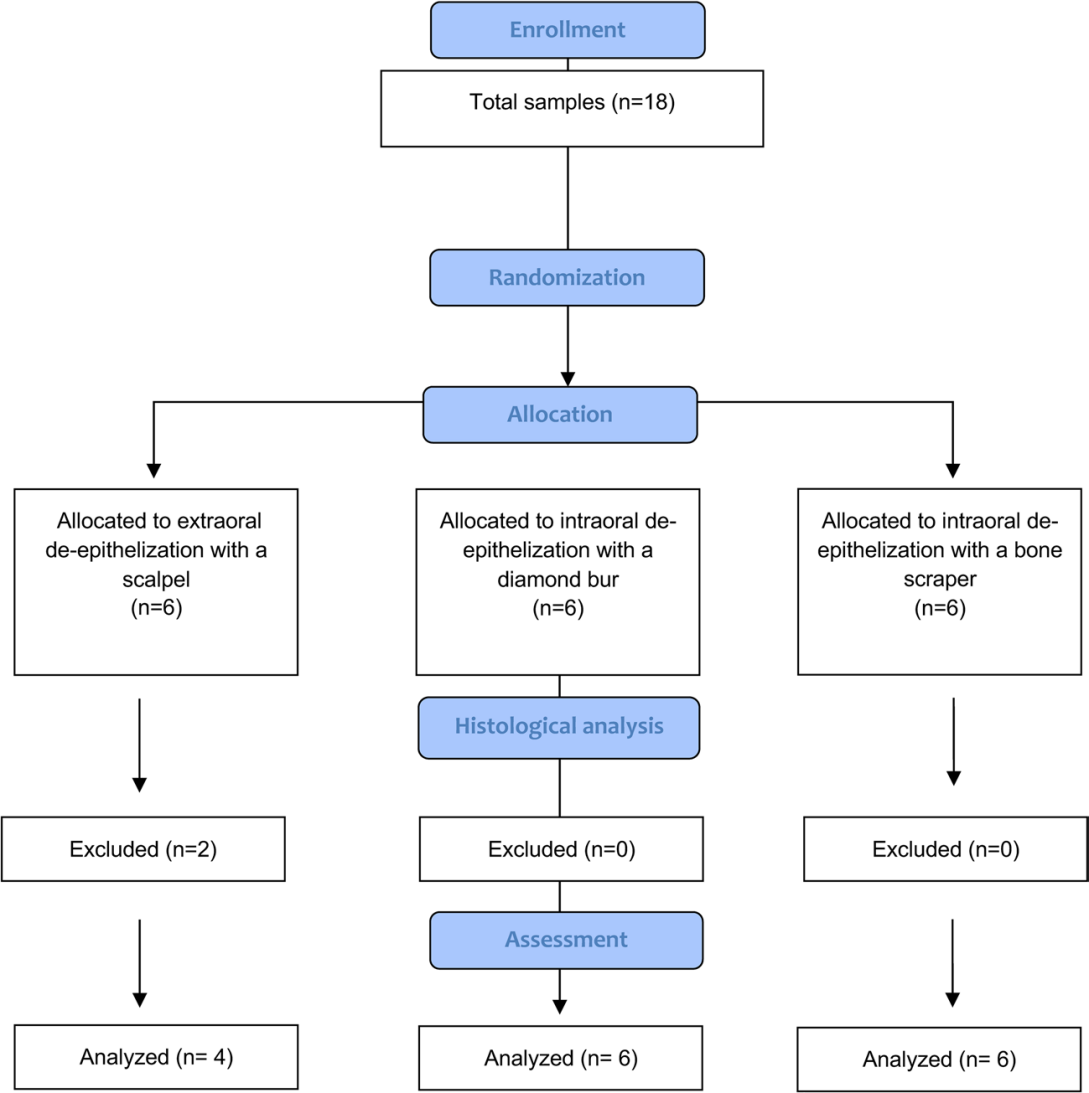
Flowchart for the study

Finally, a total of 16 samples were histologically analyzed due to deterioration of two samples during the histological analysis. The flow chart for the present study is shown in Fig. 2.

**Fig. 3:**
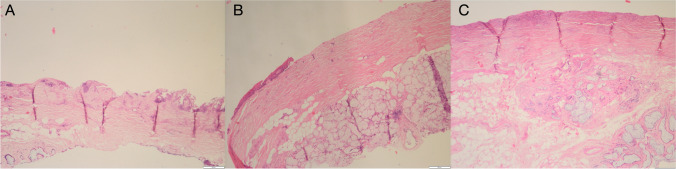
Histological images of the de-epithelialization techniques. **A**. Extraoral de-epithelialization with scalpel (H&E × 20); **B**. Intraoral de-epithelialization with a diamond bur (H&E × 20); **C**. Intraoral de-epithelialization with a bone scraper (H&E × 20). H&E: Hematoxylin–eosin stain

**Fig. 4:**
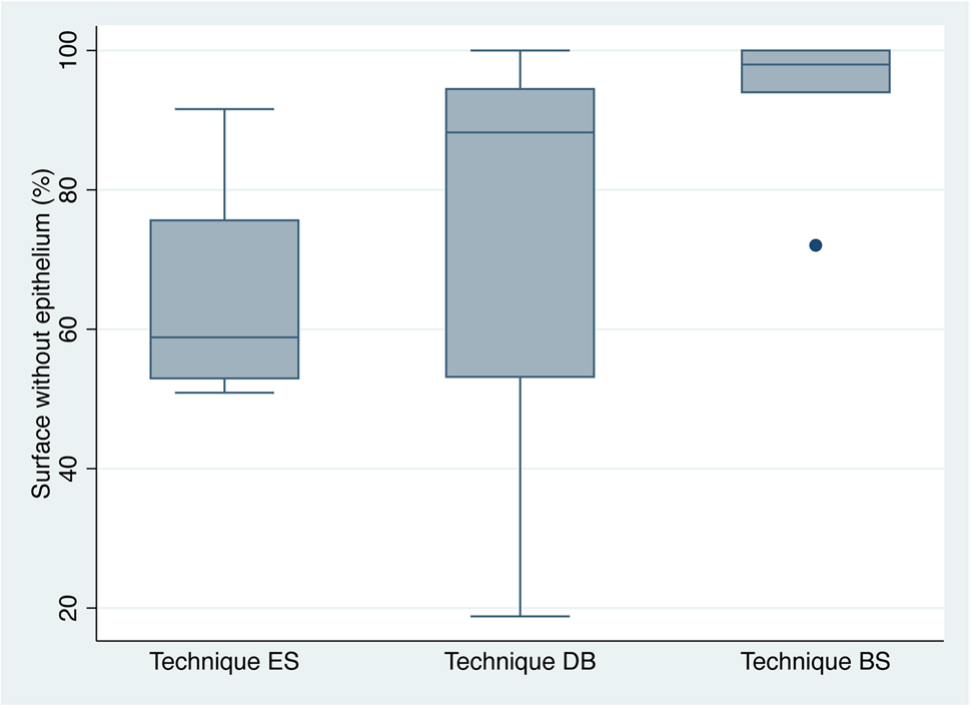
Box plots summarizing the results of the study. ES: Extraoral de-epithelialization with scalpel; DB: Intraoral de-epithelialization with a diamond bur; BS: Intraoral de-epithelialization with a bone scraper

The extraoral group presented a mean total surface area without epithelium remnants of 58.84% (22.68). On the other hand, the diamond bur and bone scraper groups had better outcomes in terms of de-epithelialization, with a mean total surface without epithelium of 88.24% (41.3) and 97.975% (5.99), respectively. Indeed, only one CTG in the bone scraper group had more than 5% of epithelium remnants. Significant differences were found between the bone scraper group and the extraoral group (*P* = 0.009). The other comparisons (extraoral Vs. diamond bur (*P* = 0.393) and diamond bur Vs. bone scraper (*P* = 0.226)) did not yield significant differences. All samples of the extraoral group had some amount of epithelium coverage, while 1 sample (25%) of the DB group and 3 samples (50%) of the BS group were free of epithelium (*P* = 0.131). Results are summarized in Table 1 and Figs. 3 and 4.

**Table 1:** Summary of results of the de-epithelialization techniques

	Surface without epithelium (in percentage, %) Median (IQR)	Total samples without epithelium N (%)	Grafts thickness (in mm) Median (IQR)	Epithelium thickness (in mm) Median (IQR)	De-epithelialization time (in minutes) Median (IQR)
Technique ES	58.84 (22,68)	0 (0%)	1.41 (0.33)	0.21 (0.17)	3.7 (0.15)
Technique DB	88.24 (41,3)	1 (25%)	1.75 (0.55)	0.23 (0.19)	1.455 (0.3)
Technique BS	97.975 (5,99)	3 (50%)	1.78 (0.65)	0.06 (0.11)	0.815 (0.28)
*P* value	0.039*	0.131	0.245	0.202	< 0.001*

*N* number of samples; *ES* extraoral de-epithelialization with scalpel; *DB* intraoral de-epithelialization with a diamond burr; *BS* intraoral de-epithelialization with a bone scraper; *IQR* Interquartile range, mm: millimeters. * *P* value is significant (*P*<0.05)

Significant differences were also found regarding the time needed to perform the de-epithelialization of the graft (*P* < 0.001). The bone scraper technique was the fastest with a mean required time of 0.815 min (0.28), whereas the extraoral technique was the slowest (mean time of 3.7 min (0.15) (Table 1*)*.

When evaluating graft thickness, no statistically significant differences were observed among the groups (*P* = 0.245). The thinnest graft thickness was associated with Technique ES at 1.41mm (0.33), while the thickest was associated with Technique BS at 1.78mm (0.65). Additionally, the thinnest epithelium remnants were found with Technique BS, with a median maximum of 0.06mm (0.11), while the median thickest remnants were recorded with Technique DB at 0.23mm (0.19). However, there were no significant differences within groups (*P* = 0.202).

## Discussion

This study aims to evaluate a novel technique for CTG de-epithelialization and compare it with other previously described options [1, 4, 8, 12]. To the authors knowledge, the use of a bone scraper as a de-epithelialization device has not been described previously and, within the limitations of the present study, it seems that a bone scraper can be a useful tool to perform the de-epithelialization of CTGs, as it is easier, faster and more effective than the commonly-used techniques.

The concept behind using a bone scraper for de-epithelialization is very similar to the dermatome skin graft devices that are able to remove a superficial layer of tissue of a fixed width. Back in 1936, these devices were introduced in plastic surgery, specifically to treat burned patients. This method was considered a fast and easy way to obtain free skin grafts [15–17]. Nowadays, there is a wide variety of dermatome skin graft devices but all of them aim to remove a fragment of skin of a constant thickness from the donor site.

Recently, several studies compared various de-epithelialization methods. Couso-Queiruga et al. [4] found no significant difference between de-epithelialization using a ball bur (intraoral method) or a scalpel (extraoral method). On the other hand, Sebaoun et al., stated that the use of a diamond ball bur seemed to be the best option [12]. Several authors [13, 18] have concluded that performing an extraoral de-epithelialization with a 15C scalpel seem not to be entirely effective. Indeed, Maia et al. obtained similar findings to the ones reported in the present study concerning the presence of epithelium remnants (44.32% Vs. 41.16% of the present study) [13].

Other de-epithelialization techniques such as diode laser or the intraoral trap-door technique [5, 19] have been described in the literature. Ozcelik et al. stated that epithelial removal with laser is as effective as the conventional blade but has the advantage of providing a better postoperative course [19] and less esthetic impairment [5].

Focusing on the results of the treatment of gingival recession using a CTG, recent studies reported that the use of CTG via coronally advanced flap (CAF) obtained either with the extraoral de-epithelialized gingival graft or the traditional trap door technique had the same outcomes in terms of root coverage and clinical attachment [5].

De-epithelialization techniques should focus on eliminating the epithelium but avoiding removing the lamina propria and entering the submucosa, which would result in lower density of collagen [4, 5, 7]. Therefore, a device that controls the depth of tissue removal might be very interesting. The bone scraper seems to be an interesting option since it removes approximately 0.25mm of tissue with each use (Safescraper, curved twist model, META, Reggio Emilia, Italy).

In the present study, all samples were collected from the posterior palatal area. Keratinized mucosa was found to be significantly thicker in the tuberosity and in the posterior zone of the palate [20] and the subepithelial tissue from the tuberosity was found to be dense and to have less adipose and glandular tissue [6]. Hence, the best donor site from a histological point of view seems to be the tuberosity, because it has a thick lamina propria without the presence of a smaller submucosal layer [6, 21]. Nevertheless, surgical access to this area is difficult and the quantity and shape of connective tissue might be inadequate in some circumstances [22].

In a clinical setting, de-epithelialization can be intraoperatively assessed because bleeding occurs since the epithelial layer does not contain blood vessels. In the present study, this sign could be considered a limitation because the samples were taken from cadaver specimens, but also in a clinical situation this could lead to false positives, since the blood can be spilled to other areas of the sample without removing epithelium and generate confusion [4].

Zucchelli et al., found that a coronally-advanced flap (CAF) with CTG de-epithelialized extraorally was significantly faster than the traditional trap door technique (35.8 ± 3.4 min Vs. 45.0 ± 4.3 min) [5]. Time is an important variable to consider, since it is desirable to reduce the extraoral period in which the graft does not receive any type of vascularization [12]. Thus, de-epithelialization with a bone scraper seems to be an excellent choice.

The novel de-epithelialization method presented in this study could be an interesting option for novice surgeons, thanks to its simple handling. However, this technique has more associated costs since a disposable scrapper is needed. Future research should analyze if conventional metallic bone scrappers can obtain a similar outcome and if the surgeon’s experience has an impact in the results of these techniques.

Some limitations should be considered when analyzing the present study. Firstly, the small sample size could limit the external validity of the outcomes. Secondly, the oral mucosa had different consistencies and different thicknesses among the specimens, even though all cadavers underwent the same cryopreservation methodology. The main outcome of the present study was the effectiveness in de-epithelialization of the grafts, while the composition of the samples (i.e., lamina propria/submucosa) was not evaluated. For this reason, future research should analyze the composition of the grafts harvested and the success of the graft in the receiving area using the various techniques. Large sample randomized controlled clinical trials (RCT), comparing the different available techniques, and including other outcome variables such as patient-reported outcome measures (PROMs), global satisfaction and quality of life impact are needed to confirm the present findings.

In conclusion, and taking into consideration the limited sample size of this study, de-epithelialization with a bone scraper seems to be a simple, fast and effective technique to remove the epithelium of CTGs. Additionally, the de-epithelialization technique of palatal CTG with a bone scraper appears to be faster than the rest of the techniques described.

## Data Availability

The data that support the findings of this study are not openly available due to reasons of sensitivity and are available from the corresponding author upon reasonable request.
